# Effects of dominance and female presence on secondary sexual characteristics in male tufted capuchin monkeys (*Sapajus apella*)

**DOI:** 10.1002/ece3.7483

**Published:** 2021-03-30

**Authors:** Annika Paukner, Emily M. Slonecker, Lauren J. Wooddell

**Affiliations:** ^1^ Department of Psychology Nottingham Trent University Nottingham UK; ^2^ Department of Psychological Science University of California Irvine Irvine CA USA; ^3^ Yerkes National Primate Research Center Field Station Lawrenceville GA USA

**Keywords:** alpha male, face, secondary sexual characteristics, testes, weight

## Abstract

Alpha status may lead to physiological changes that enhance secondary sexual characteristics, which may serve as competitive signals to conspecific males, sexual signals to females, or possibly a combination of both. Here, we report measurements of secondary sexual characteristics in captive dominant and subordinate male tufted capuchin monkeys (*Sapajus apella*) with varying access to females. An adult male (who had previously been subordinate while housed with other males) was paired with an adult female. This male–female pair was introduced into a room that housed three other male–male pairs with stable hierarchy arrangements. We analyzed weight, body measurements, facial photographs, and hair cortisol before, during, and after introducing a female into the room. While there were no differences in weight or measurements between alphas and subordinates without physical access to the female prior to or during the female's presence, we found that direct access to the female resulted in dramatic changes in facial appearance, body size, and testicular volume in the male who was paired with her. Overall, we found little evidence to suggest that alpha males advertise their status within all‐male groups via sexual secondary characteristics. However, direct physical access to females appears to trigger the development of such characteristics in alpha males. It remains of continued interest to identify the endocrine mechanisms responsible for the development, and possible loss, of secondary sexual characteristics.

## Graphical abstract

We investigated whether secondary sexual characteristics in male tufted capuchin monkeys were related to dominance rank or presence of a female. Our results suggest that the presence of a female can lead to the development of secondary sexual characteristics in alpha males, but that dominance status alone may not be sufficient.

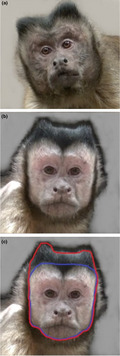

## 1 INTRODUCTION

Studies of development frequently focus on how infants and juveniles mature and become adults, a milestone frequently demarcated by reaching reproductive age (McNamara, 2004). However, in many species, including humans, reproductive capability is often reached before physical development is complete (Daly & Wilson, 1983). Reproductively capable individuals may lack species‐typical secondary sexual characteristics, such as male orangutans (*Pongo spp*.) that remain unflanged (Utami et al., 2002). In some species, sexual secondary characteristics may develop rapidly within just a few months (e.g., final growth spurt in male long‐tailed macaques, *Macaca fascicularis*; Van Noordwijk & van Schaik, 2001). Some of these physiological changes in males appear to be linked to reproduction itself and thus triggered by environmental cues, either seasonally or through female behavior (e.g., zebra finches, *Taeniopygia guttata*; Gautier et al., 2008). In group‐living species, increases in dominance rank, and in particular achievement of alpha male status, which is typically linked to increased reproductive opportunities, may lead to physiological changes such as increases in testicular volume and fattedness (e.g., mandrills, *Mandrillus sphinx*; Setchell & Dixson, 2001), as well as development of specific adornments (e.g., blue scrotal color in vervet monkeys, *Chlorocebus pygerythrus*, Gerald, 2001; reddening of the sexual skin on the face and genitalia of mandrills, Setchell & Dixson, 2001; stained chests in Verreaux's sifaka, *Propithecus verreauxi*, Lewis & van Schaik, 2007; redness of gelada baboon chest, *Theropithecus gelada*, Dunbar, 2014). Loss of alpha status may reverse some of these status indicators (mandrills, Setchell & Dixson, 2001; geladas, Dunbar, 2014; sifakas, Lewis & van Schaik, 2007; see also Georgiev et al., 2016, for effects of loss of alpha status in a male rhesus macaque).

Changes in appearance may serve as a signal of alpha status to females (intersexual selection) or to other males within the group (intrasexual selection), or potentially both. As sexual signals tend to mirror the health status of the bearer (Lozano, 1994), females may be able to assess a male's genetic quality (e.g., peafowls, *Pavo cristatus*; Petrie, 1994), while males may avoid physical fights that are costly to both parties (e.g., rhesus macaque, *Macaca mulatta*; Petersdorf et al., 2017; mandrills, Setchell & Dixson, 2001). The utility of signals seems to be more beneficial between individuals who are largely unknown to each other (Bergman et al., 2009; Setchell & Jean Wickings, 2005), but signals can also occur in species where individuals have a long history of prior interaction (e.g., wahoo calls in baboons, *Papio cynocephalus ursinus*; Kitchen et al., 2003) and in large socially complex groups (Grueter et al., 2015). A signal that is costly for a male to produce or maintain may be downregulated in the absence of a receiver, that is, females in the case of intersexual selection or males in the case of intrasexual selection (Gautier et al., 2008).

**FIGURE 1:**
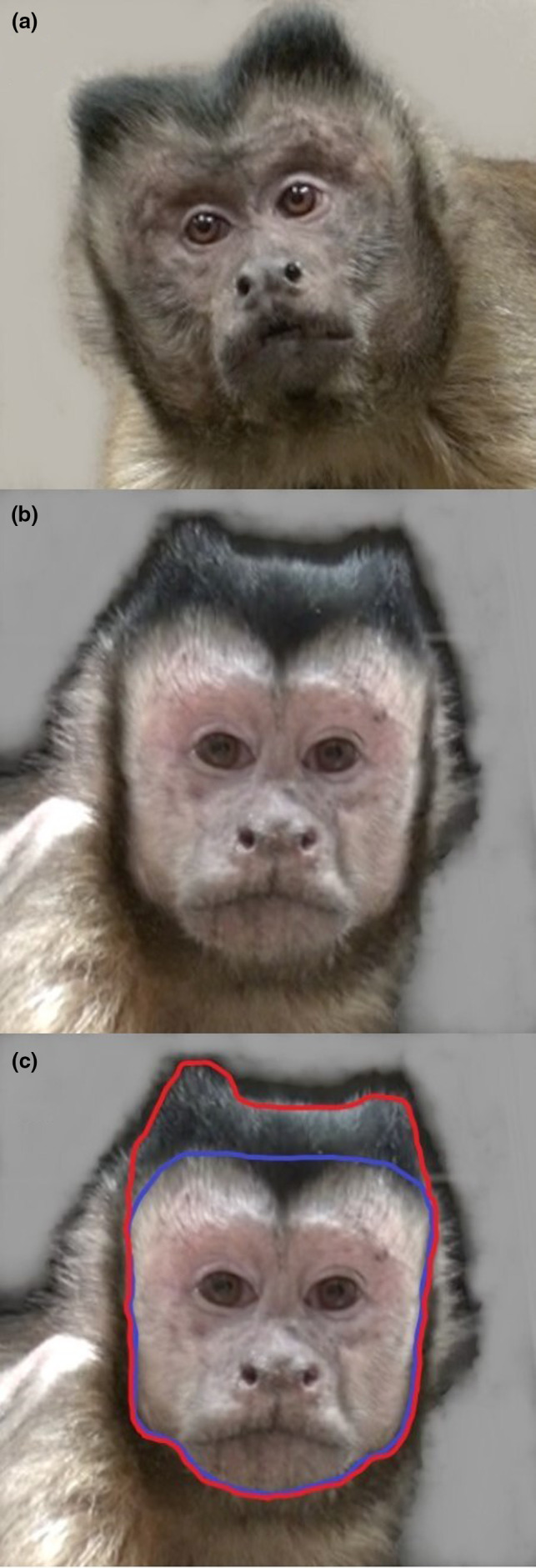
Male tufted capuchin monkeys (*Sapajus apella*). (a) 12‐year‐old alpha male housed with a female (JR, photo from 2016). (b) 12‐year‐old subordinate male housed with another male (MR, photo from 2016). (c) Outline of indicative facial measurements. Blue line—face measurement, red line—head measurement

Tufted capuchin monkeys (S*apajus apella*; Figure 1) are moderately sexually dimorphic with males exceeding females in body mass (Fragaszy et al., 2004). Physical features of male capuchins may change as a consequence of achieving alpha status, although this has only been observed anecdotally. For instance, alpha male bearded capuchins (*Sapajus libidinosus*) are described as bulkier in the head and forequarters than other males (Fragaszy et al., 2016), and in white‐faced capuchins (*Cebus capucinus*), males are described as developing an exaggerated brow ridge and a widened mandibular girth after they achieve alpha status, making alpha status a distinct life‐history stage (Jack et al., 2014) with keystone functions (Jack & Fedigan, 2018). Since female capuchins actively solicit males for mating (Alfaro, 2005; Falótico & Ottoni, 2013) with the alpha male being the preferred mating partner in approximately 70% of matings (Fragaszy et al., 2004), distinct appearance may be one of several possible signals that could advertise alpha status to females. Although capuchin males rarely compete directly with other males over copulations with females, they compete indirectly by vying for alpha rank within a group (Schaebs et al., 2017). Appearance could therefore also be a useful signal for other males, leading to low in‐group male–male aggression in capuchins (Lynch et al., 2002). In addition, male bearded capuchins gain body mass after they achieve alpha status and may lose body mass when alpha position is lost (Fragaszy et al., 2016). It is less clear whether this change in body mass is a consequence of physiological changes associated with achieving alpha status (such as an increase in testosterone production; Mendonça‐Furtado et al., 2014) or whether it is a consequence of the benefits of being alpha (e.g., being able to monopolize food sources; Di Bitetti & Janson, 2001).

While anecdotal evidence suggests that alpha capuchin monkeys develop secondary sexual characteristics, empirical work on this topic is limited and it remains unclear whether these changes are partially explained by the social benefits of alpha status (e.g., increased food access). Here, we sought to determine whether alpha male status in tufted capuchin monkeys is associated with distinct secondary sexual characteristics, and whether the expression of these characteristics is dependent on the presence of an adult female, by measuring the physical condition and facial appearance of captive male tufted capuchin monkeys with varying access to a female. The current study was conducted with the tufted capuchin monkey colony at the Laboratory of Comparative Ethology, National Institutes of Health Animal Center near Poolesville, Maryland, USA, which experienced changes to their housing arrangements necessitated by facility management decisions. These changes resulted in an adult female being paired with an adult male (JR), who was previously low‐ranking in an all‐male group. The newly established male–female pair was housed in a colony room with visual, auditory, and olfactory contact to three other male–male capuchin pairs with stable dominance relationships. JR therefore became the only male to have direct access to the female while all other males had visual/auditory/olfactory contact, but no physical contact, with the female. As part of a longitudinal, unrelated research project, we had collected physical measurements and facial photographs of all male monkeys prior to this change in housing arrangements. We thus used these measurements to retrospectively document physical changes exhibited by JR following his pairing with the female and to explore how these changes compared to the physical characteristics exhibited by the other males (both alpha and subordinate). Since food and other environmental factors were held constant throughout the study period, any changes in physical appearance in JR can be attributed to changes in the social environment, not to a secondary effect of access to higher quality or quantity of food.

We hypothesized that males advertise their alpha status primarily to other males, based on previous findings that male, but not female, tufted capuchins are sensitive to facial symmetry in other male capuchins (Paukner et al., 2017), and male capuchins spent more time looking at pictures of male capuchin faces compared to female capuchin faces (Lonsdorf et al., 2019). However, the presence of a female might further enhance the expression of secondary sexual characteristics, especially in alpha males who might gain mating opportunities. These gains could be dependent on the presence of the female, that is, without female presence and potential mating opportunities, alpha males' secondary sexual characteristics could be costly to maintain and might revert to previously seen levels. We were also able to gain insights into some of the underlying physiology of these changes by analyzing glucocorticoids in hair samples collected after the introduction and later removal of the female.

We predicted that:

1. Prior to the introduction of a female, alpha males housed in same‐sex pairs would be bigger (in weight, facial measurements, body measurements) than subordinate males.
2. Introduction of a female would result in increased weight, face measurements, and body measurements in all alpha males. However, the male with direct access to the female (JR) would show a significantly larger increase in measurements relative to the males without direct access.
3. If alphas experienced a gain in their weights, measurements, and facial appearance following the introduction of the female, then removal of the female might reverse some of these gains.

Unfortunately, we were only able to investigate the last prediction in the same‐sex pairs as JR remained housed with the female for the duration of the study.

## 2 METHODS

All data were collected at the Laboratory of Comparative Ethology, National Institutes of Health Animal Center, near Poolesville, Maryland, USA, between April 2012 and April 2016. All applicable national and institutional guidelines for the care and use of animals were followed. All procedures were in accordance with the ethical standards of the institution at which the studies were conducted (*Eunice Kennedy Shriver* National Institute of Child Health and Human Development Animal Care and Use Committee, ASP #12‐015 and #15‐015).

### 2.1 Subjects

We studied seven males (aged between 5 y1 m and 9 y9 m in April 2012; Table 1) in conjunction with one adult female (aged 12 y9 m in April 2012). All monkeys were born and reared in captive social groups. All monkeys were housed indoors for the duration of the study and received their regular diet of commercial monkey biscuits (Purina Monkey Chow, #5054, St Louis, MO) as well as twice‐daily enrichment (scatter feed of grains or seeds in the mornings, fruit or nuts in the afternoon). All monkeys received a set amount of monkey biscuits per group, determined by the number and size of monkeys in each group. It was facility practice that at feeding time, a small amount of monkey biscuits from the previous feeding should still be present in the cage, thus ensuring that all monkeys had plentiful access to food. Water was available ad libitum.

**TABLE 1:** Overview of ages, rank, and housing arrangements of monkeys

Monkey	Group	Rank	Date of birth	Age at start of study in 2012	Age when female was introduced in 2014
IC	1	Alpha	April 2006	6 years	7 years 10 months
JA	1	Subordinate	November 2006	5 years 5 months	7 years 3 months
SH	2	Alpha	March 2003	9 years 1 month	10 years 11 months
LE	2	Subordinate	March 2007	5 years 1 month	6 years 11 months
HO	3	Alpha	July 2002	9 years 9 months	13 years 7 months
MR	3	Subordinate	October 2004	7 years 6 months	9 years 4 months
JR	4	Subordinate ‐> alpha (paired with female)	May 2004	7 years 11 months	9 years 9 months

At the start of the study, monkeys were housed in two pairs (Group 1: IC, JA; Group 2: SH, LE) and one group of 3 (HO, MR, JR) in the same room with visual/auditory/olfactory access to each other. All monkeys came from multi‐male/multi‐female social groups, and none were alpha males prior to being housed in all‐male groups. Dominance ranks were evident as observed through displacements and other dyadic aggression within groups and remained stable for all pair‐housed monkeys for the duration of the study. For the group of 3 monkeys, the two lower‐ranking monkeys (JR, MR) switched dominance ranks several times during the first data collection phase prior to introduction of the female; HO remained the highest‐ranking monkey of the group throughout.

In February 2014, facility management decisions necessitated that the then lowest‐ranking monkey (JR) was paired with an adult female from another group (LY). JR was removed from his group and successively introduced (visual contact only at first, then contact through a mesh panel, then full contact) to LY in another room without any visual/auditory/olfactory contact with other monkeys. After approximately 2 weeks, they were moved back into the room where the other male pairs were housed. Thus, there were three male pairs (Pair 1: IC, JA; Pair 2: SH, LE; Pair 3: HO, MR) and one male–female pair (Pair 4: JR, LY), each with a stable dominance hierarchy (dominant monkeys were IC, SH, HO, and JR). Each pair had visual/auditory/olfactory access, but not physical access, to the other pairs. To avoid unintended pregnancies, JR was vasectomized in April 2014. LY was treated with the injectable contraceptive medroxyprogesterone acetate (MPA; Depo‐Provera, 20 mg/kg) between February and May 2014 and cycled naturally thereafter until July 2015, when she was put back on to Depo‐Provera. In November 2015, facility management decisions resulted in JR and LY being moved to another building; the three male pairs remained housed together in the same room (but in separate enclosures).

### 2.2 Procedure

We collected body measurements, weights, and facial photographs from all monkeys during routine quarterly veterinary health examinations from 2012 to 2016. We were able to collect hair samples between 2014 and 2016. All monkeys were sedated with a 10:1 mixture of ketamine and acepromazine at 0.1 ml/kg (9 mg/kg ketamine and 0.1 mg/kg acepromazine), IM. We collected body measurements and weights on eleven occasions (three prior to introduction of the female, six while the female was present in the room, two after removal of the male–female pair), facial photographs on ten occasions (two prior to introduction of the female, six while the female was present in the room, two after removal of the male–female pair), and hair samples on 6 occasions (four while the female was present in the room, two after the removal of the male–female pair).

#### 2.2.1 Body measurements

We collected a range of body measurements from monkeys. Some measurements were related to soft tissue (testicle size, circumference, skinfold), and therefore, we hypothesized these measurements could be amenable to changes in the social environment. Other measurements were based on hard tissue (bone lengths), and while it is possible that certain social circumstances may trigger an increase in these measurements, they are less likely to show decreases based on social factors. Measurements included crown‐rump length (top of the skull to the most distal part of the ischial tuberosity), ulna length (wrist bone to end of ulna with elbow bent), femur length (top of femur to tip of bent knee), neck circumference (narrowest part), chest circumference (just below nipples), abdominal circumference (at umbilical level), biceps circumference (widest part with elbow bent), scapula skinfold (1 cm below scapula), upper abdominal skinfold (2 cm above umbilicus), lower abdominal skinfold (2 cm below umbilicus), foot length (distal end of calcaneus to tip of third digit), and testicle size (one testicle isolated in upper portion of scrotum and compared to testicle mass while scrotal skin stretched taut). All measurements were made on the right side with the monkey in left lateral recumbency. Body and body‐segment lengths were measured with sliding calipers (in cm). Circumferences were measured with a tape measure to the nearest 0.5 cm. Skinfold was measured using medical skinfold calipers (in mm). Testicle size was estimated using an orchiometer (in ml). Each measurement was taken twice by the same experimenter (AP) and had to be within 0.5 measurement units of each other; an average of both measurements was used for analysis.

#### 2.2.2 Photographs

Facial photographs were collected with a digital video camera (Sony HDR‐CX560V). All pictures were frontal and included the face and all hair tufts. Prior to measurement, photographs were aligned and scaled according to interpupilar distance. Since monkeys were sedated (eyes closed) when pictures were taken, we determined interpupilar distance following Pryor (1969) by measuring the distance between the outer angles of the eyes (a), the distance between the inner angles of the eyes (b), and calculating (A − B)/2 + B. Photographs were then uploaded into Tobii Studio (Tobii Technology) and pixel sizes of the face and the head were measured by drawing areas of interest (AOIs). The head measurement consisted of the face area (from jaw line to top of forehead) as well as the hair tufts on top of the head, and the face measurement consisted of the just face area without any hair tufts (Figure 1). A subset of photographs (*N* = 16, 20%) were measured by a second coder, and reliability measures were good (ICC = 0.99 for full head, 0.80 for face). Both coders were blind to the hypotheses under investigation at the time of measurement.

#### 2.2.3 Hair cortisol analysis

Hair was shaved from the back of the neck following a standardized protocol developed for rhesus macaques (Davenport et al., 2006). Samples were placed in an aluminum foil pouch and stored in a −80°C freezer. Hair samples were weighed, washed twice with isopropanol, allowed to air dry for 5–7 days, then ground to a fine powder, and incubated in methanol for 18−24 hr. Aliquots of the methanol extract were then dried down and reconstituted with assay buffer at a dilution of 1:8 prior to being analyzed via enzyme immunoassay (EIA) using a salivary cortisol kit (#1‐3002; Salimetrics, State College, PA). Resulting values (μg/dl) were converted to pg/mg for analysis. Intra‐assay coefficients of variation were <10%.

## 3 RESULTS

1. Prior to the introduction of a female, alpha males housed in same‐sex pairs would be bigger (in weight, facial measurements, body measurements) than subordinate males

**FIGURE 2:**
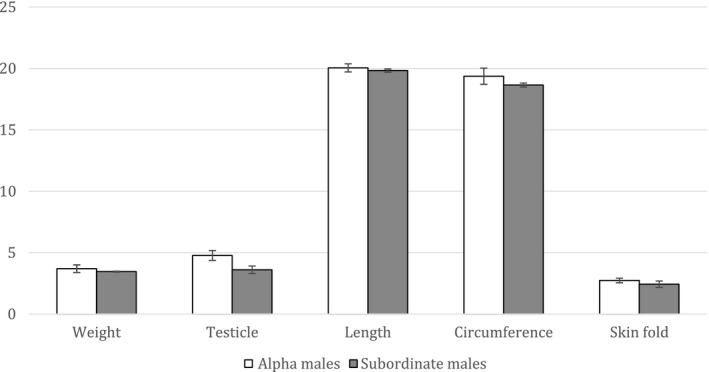
Summary of average weight and body measurements of alpha and subordinate males in the absence of any females. Length = average of crown‐rump, ulna, femur, and foot measurements, circumference = average of neck, chest, abdomen, and biceps measurements, skinfold = average of scapula, upper abdomen, and lower abdomen measurements. Error bars depict standard errors. Weight in kg, testicle size in ml, length and circumference in cm, skinfold in mm

We used randomization tests to evaluate this prediction. We averaged measurements from 2012 to 2014 (prior to introduction of the female) for all monkeys whose dominance rank remained stable (HO, IC, JA, LE, MR, SH). In randomization tests, the proportion of 1,000 randomly sampled data divisions giving differences in the predicted direction (alpha > subordinate) at least as large as the experimentally obtained difference across weights, face measurements, and individual body measurements were *p* > .05 and therefore not statistically significant (Todman & Dugard, 2001; see Figure 2). In the absence of females, alpha males were not significantly larger than subordinate males on any individual measurement.

2. Introduction of a female would result in increased weight, face measurements, and body measurements in all alpha males. However, the male with direct access to the female (JR) would show a significantly larger increase in measurements relative to the males without direct access

The following results compare the last measurement prior to introduction of the female (January 2014) to the average measurements collected while cohoused with the female (April 2014 to October 2015). By April 2014, all males were considered full adults (ranging in ages from 7 years 1 month to 11 years 1 month).

### 3.1 Male (JR) paired with female

1. Weight

JR increased his weight from an average of 3 kg prior to introduction of the female to an average of 3.92 kg after introduction of the female, a gain of 30.56%.

2. Body measurements

Most of JR's body measurements increased modestly but consistently after being paired with the female. His limb measurements showed an average increase of 2.65%, and his circumference measurements showed an average increase of 14.71%. JR also gained in body fat with his skinfold measurements increasing on average 60.41%. The most dramatic change was observed in testicle size from 3 prior to introduction to an average of 11.33 after introduction, an increase of 277.78%.

3. Face measurements

Facial measures also showed an increase after introduction of the female: on average, the head size (head and tufts) increased by 12.78% and face size increased by 17.53%.

### 3.2 Alpha males not paired with female (HO, IC, SH)

1. Weight

On average, the established alpha males only gained a modest amount of weight following the introduction of the female to the room (averages from 3.6 kg to 3.79 kg, +5.4%).

2. Body measurements

The established alphas' body measurements showed no consistent pattern of gain or loss. On average, alphas lost −0.98% in limb measurements, −1.04% in circumference measurements, and gained 11.46% in skinfold measurements. Testicle measurements showed a small increase from 5.00 to 5.06, or 1.11% increase.

3. Face measurements

Face size of alpha males slightly changed after introduction of the female into the room. On average, head size decreased by 2.70% and face size increased by 0.22%.

### 3.3 Subordinate males not paired with female (JA, LE, MR)

1. Weight

Weight of subordinate males slightly increased after introduction of the female into the room, from an average of 3.48 kg to 3.53 kg, or 1.44%.

2. Body measurements

Subordinate monkeys' body measurements showed a slight increase in limb measurements (average 0.14%) but decreases in circumference measurements (average −0.47%) and skinfold measurements (average −6.09%). Testicle sizes showed an increase from averages 3.67 to 3.82, or 4.28%.

3. Face measurements

Facial measurements showed inconsistent changes after introduction of the female into the room. Head size increased by an average of 2.48% and face size decreased by an average of 1.44%.

### 3.4 Comparison of physical changes between alpha and subordinate males

**FIGURE 3:**
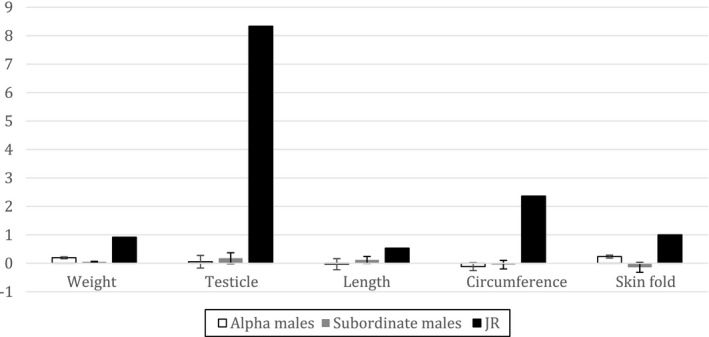
Summary of average change in weight and body measurements of alpha males, subordinate males, and JR after being cohoused with the female. Length = average of crown‐rump, ulna, femur, and foot measurements, circumference = average of neck, chest, abdomen, and biceps measurements, skinfold = average of scapula, upper abdomen, and lower abdomen measurements. Error bars depict standard errors. Weight in kg, testicle size in ml, length and circumference in cm, skinfold in mm

We used randomization tests to evaluate this prediction. We calculated an average difference score between the last measurement prior to introduction of the female (January 2014) and the average measurements taken while cohoused with the female (April 2014–October 2015) for all alpha males (IC, SH, HO) and subordinate males (JA, LE, MR). In randomization tests, the proportion of 1,000 randomly sampled data divisions giving differences in the predicted direction (alpha > subordinate) at least as large as the experimentally obtained difference across weights, face measurements, and individual body measurements were *p* > .05 and therefore not statistically significant (Todman & Dugard, 2001; Figure 3). Alpha males did not gain significantly more in size or facial appearance compared to subordinates.

### 3.5 Comparison of physical changes in JR to all other males

To assess whether the changes observed in JR were significantly different from the changes observed in the males housed in same‐sex pairs, we calculated an average difference score between the last measurement prior to introduction of the female (January 2014) and the average measurements taken while cohoused with the female (April 2014–October 2015) for each monkey and each measure. We then converted JR's measurements into *z*‐scores using the means and standard deviations of the males (*n* = 6) housed in same‐sex pairs to establish population means and standard deviations. Using two‐tailed tests (*α* = 0.05), JR gained significantly more weight (*z* = 8.84, *p* < .0001), testicle size (*z* = 24.96, *p* < .0001), neck circumference (*z* = 4.83, *p* < .0001), chest circumference (*z* = 5.22, *p *< .0001), abdominal circumference (*z* = 2.90, *p *= .004), and bicep circumference (*z* = 4.31, *p* < .001), as well as scapula skinfold (*z* = 4.00, *p *< .001), upper abdominal skinfold (*z* = 2.18, *p *= .029), and lower abdominal skinfold(*z* = 1.97, *p *= .049) than the other males housed in same‐sex pairs. In addition, comparisons in facial measurements showed that JR's face size (*z* = 2.02, *p* = .044) increased significantly more than those of the males housed in same‐sex pairs. Similar results were obtained when comparing JR's data to just the alpha males. In sum, JR's physical gains were larger than those of the other males, most notable in his face, circumference measurements, skinfold measurements, testicles, and weight (Figure 3).

3. If alphas experienced a gain in their weights, measurements, and facial appearance following the introduction of the female, then removal of the female might reverse some of these gains

**FIGURE 4:**
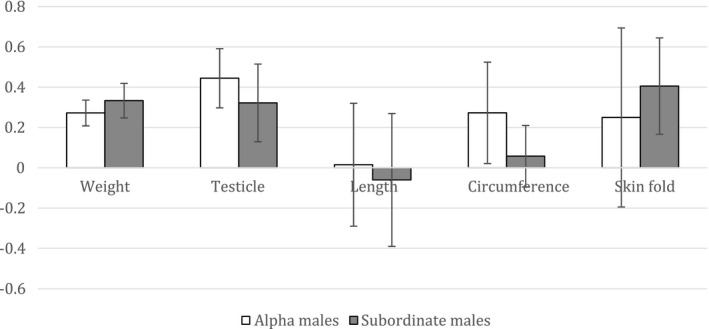
Average percentages of change in measurements in alpha males between presence of female and removal of female. Length = average of crown‐rump, ulna, femur, and foot measurements, circumference = average of neck, chest, abdomen, and biceps measurements, skinfold = average of scapula, upper abdomen, and lower abdomen measurements. Error bars depict standard errors

We compared the average from all measurements during the time the female was housed in the room (April 2014 to October 2015, six measurements) with the average from measurements taken after the male–female pair (JR‐LY) was removed from the room (January 2016 to April 2016, two measurements). We found only slight changes, with alpha males on average gaining 0.27 kg in weight, or 7.05%; increase in testicle size by 0.44, or 5.44%; 0.25% loss in length measurements; 1.26% gain in circumference measurements; and 9.18% gain in skinfold measurements. Head sizes increased by 1.22% and face size increased by 1.23% on average (Figure 4). These changes were small and since they indicate gains rather than the decreases that we expected, we did not test for statistical significance, as it is obvious that the hypothesis is not supported.

### 3.6 Hair cortisol values

**FIGURE 5:**
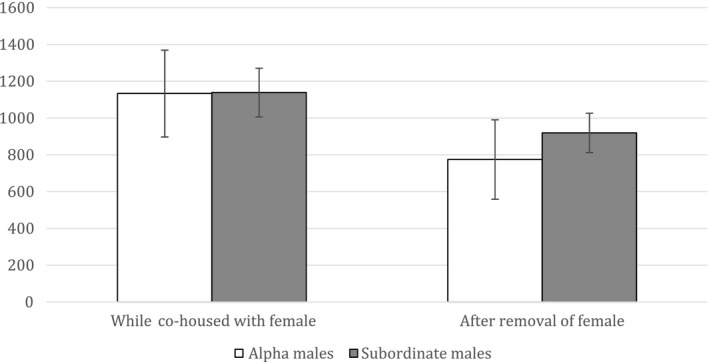
Hair cortisol values (in pg/mg) for alpha and subordinate males while cohoused with the female and after removal of the female

We unfortunately did not have hair samples prior to the introduction of the female, as hair collection for an unrelated longitudinal study began in October 2014. Hair cortisol values for all monkeys ranged between 532.48 and 2,755.02 pg/mg. While the female was housed within the same room, the three alpha males' hair cortisol levels were similar to the three subordinate males' hair cortisol levels (1,134 pg/mg vs. 1,139 pg/mg, respectively; Figure 5). During the same period, JR's cortisol levels averaged 1,678.93 pg/mg. Converting JR's cortisol values into *z*‐scores using the means and standard deviations of the other males (*n* = 6) as population means and standard deviations and using two‐tailed tests (*α* = 0.05), we found that JR's average hair cortisol value was not significantly different from the other males' hair cortisol values (*z* = 1.83, *p* > .05). However, all males' hair cortisol values dropped after JR and the female were removed from the colony room; this drop was significantly different from 0 on a one‐sample *t* test (two‐tailed, *t*(5) = 5.12, *p *= .004). We further compared hair cortisol values after the removal of JR and the female between alphas and subordinates, as well as the magnitude of decrease in cortisol values between alphas and subordinates. However, none of these comparisons showed any significant differences (*p*s > 0.05).

## 4 DISCUSSION

In this study, we measured physical appearance in relation to dominance rank in captive male tufted capuchin monkeys before, during, and after the introduction of a female. We hypothesized that secondary sexual characteristics related to alpha status would primarily serve as a signal to other males, not females; we therefore expected that alpha males would be larger than subordinate males when housed in all‐male groups. However, we anticipated that the presence of a female would further enhance secondary sexual characteristics, particularly for the male who had direct physical access to her. We also predicted that, due to energetic costs, gains achieved as a result of female presence would be lost again after the female was removed.

Contrary to our hypothesis, we did not find that alpha males were generally larger than subordinate males prior to the introduction of the female, suggesting that alpha status on its own may not be sufficient to lead to the development of secondary sexual characteristics in male capuchin monkeys. Two of the studied male pairs showed consistent differences in the predicted direction for weight and body measurements, but the third pair did not. However, it is worth noting that in the two former pairs, the alpha males had likely reached adult body mass (being at least 9 years old at the start of the study) whereas for the latter pair, the alpha might not have (being 6 years old at the start of the study when full maturity is generally considered 7 years old; Fragaszy et al., 2004). Two of the subordinate monkeys were also under 7 years old at the start of the study, suggesting that they may not have reached their full body size at that point. Thus, it remains possible that physical gains associated with male alpha status are further dependent on having reached adult body mass. Alternatively, it is possible that body mass may affect alpha status in that males with the potential to achieve larger body mass also more easily achieve alpha status. The causal direction of alpha status and body size is not determinable from the present data, but these ideas could be addressed in future studies using males of various ages and dominance status.

Introduction of a female had a dramatic effect on JR, the male who was paired with her and given full contact. JR showed significant gains in weight and physical appearance, in particular in the face, circumference measurements (soft tissue), and testicles, leading to an overall weight gain following her introduction. These gains were significantly larger than those experienced by the other adult males without direct access to the female, who exhibited small and inconsistent changes. During the course of the study, the female was observed sexually soliciting males from the all‐male pairs, but we did not observe any matings with JR. Thus, all males within her vicinity may have been affected by her behavioral and/or physiological cues of sexual availability, but JR, who had mating opportunities with her (whether realized or not), appears to have changed in the most dramatic fashion. In other species, female presence or behavior can directly influence male behavior, such as intensity of displays in Siamese fighting fish (*Betta splendens;* Doutrelant et al., 2001) or singing in starlings (*Sturnus vulgaris;* Gwinner et al., 2002). The presence of females can also affect physiological traits, such as facial redness in male mandrills (Setchell et al., 2008). Our results further clarify that it is not just the presence of females, but also males' direct access to them, that influence male physique. The observed changes may be the result of increased levels of testosterone, which can increase in the presence of fertile females in white‐faced capuchin monkeys (Jack et al., 2014), although other studies have reported that tufted capuchin monkey alphas do not show higher testosterone levels than other adult males (Lynch et al., 2002). Future studies that directly measure testosterone and/or dihydrotestosterone in relation to the physical presence of, and access to, females may shed further light on this issue.

Finally, we predicted that removal of the female from the room would reverse some secondary sexual characteristics in alpha males housed in same‐sex pairs. When the female was first introduced in April 2014, all males had achieved adult male status (7+ years old; Fragaszy et al., 2004). The presence of the female only inconsistently affected males housed in same‐sex pairs who did not have direct access to her, resulting in some small gains in weight but also losses in several body and face measurements. After removal of the female, alpha males modestly gained rather than lost weight (possibly as body fat as shown through skinfold measures).

Generally, it remains unclear when and how secondary sexual characteristics are maintained.

In some species, secondary sexual characteristics are strongly tied to alpha status and/or females (e.g., mandrills; Setchell & Dixson, 2001) whereas in others, secondary sexual characteristics are retained even in the absence of females (e.g., flanged orangutans; Utami et al., 2002). Future studies are required to understand whether secondary sexual characteristics signal a temporary ‘state’ or true ‘genetic quality’ (Setchell & Dixson, 2001) in capuchin monkeys.

The results of the hair cortisol measurements further suggest that, similar to rhesus macaques (Bernstein et al., 1989), the presence of a female may be a significant stressor to male tufted capuchin monkeys. During breeding season, rhesus macaque males may lose weight although they tend to regain it during the nonbreeding season (Bernstein et al., 1989), making breeding a possible source of energetic stress for males (Lynch et al., 2002). During the female's presence in the present study, alpha males' hair cortisol levels were comparable to subordinate males' levels, and JR's hair cortisol levels did not differ from those monkeys who did not have direct access to her. Unlike white‐faced capuchin monkeys, where the alpha male shows significantly higher levels of fecal glucocorticoids in the presence of fertile females (Schoof et al., 2014) or after intergroup encounters (Schoof & Jack, 2013), tufted capuchin monkey alphas and nonalpha males do not seem to differ in fecal glucocorticoid levels (Lynch et al., 2002), a finding replicated in our captive group. However, after removal of the male–female pair, hair cortisol levels significantly decreased across all monkeys housed in male–male pairs. There are two potential explanations for the observed decrease. Males may experience a significant level of frustration in the presence of a sexually receptive female, who may even sexually solicit them, but whom they cannot physically access. This frustration may then be redirected at subordinate males in the form of aggression, leading to elevated hair cortisol levels in both alpha and subordinate males. Alternatively, the mere presence of additional monkeys in the room, potentially increasing perceived population density (Dettmer et al., 2014) and agonistic intergroup interactions, may have increased hair cortisol levels. It is worth noting that actual population density did not change between study phases as the monkeys' enclosure size remained constant throughout the study. Future studies could investigate to what degree the presence of other monkeys (male or females, with and without direct physical contact to each other) affects cortisol and behavioral indicators of stress in tufted capuchin monkeys.

Overall, we found little evidence to suggest that in all‐male groups, alpha males advertise their status to other males via sexual secondary characteristics. Instead, alpha status while having direct physical access to females appears to trigger the development of such characteristics, most notable in the male's size and face. It does not necessarily follow that males' secondary sexual characteristics serve as signals primarily to females; it is equally possible that signaling to males only becomes relevant in the presence of females. Given the small sample size and opportunistic nature of the present study, the findings presented here may be considered preliminary and in need of further careful experimental investigations including more frequent, and therefore more robust, measurements of males during the different phases of the experiment. It also remains of continued interest to identify the mechanisms responsible for the development, and possible loss, of secondary sexual characteristics. Changes in endocrine function promoted by social factors have been established in relation to reproductive suppression (e.g., cotton‐top tamarins, *Saguinus oedipus*; Ziegler et al., 1995), and we strongly encourage future studies to investigate endocrine functions and behavior in relation to secondary sexual characteristics.

## CONFLICT OF INTEREST

None declared.

## AUTHOR CONTRIBUTIONS

**Annika Paukner:** Conceptualization (equal); data curation (equal); formal analysis (equal); project administration (equal); supervision (equal); writing‐original draft (equal); writing‐review & editing (equal). **Lauren J. Wooddell:** Data curation (equal); writing‐review & editing (equal). **Emily M. Slonecker:** Data curation (equal); writing‐review & editing (equal).

## Data Availability

Data associated with this manuscript can be found here: https://doi.org/10.5061/dryad.3bk3j9kh6.
